# Differential hemodynamic adaptations to tilt test in patients with idiopathic atrial fibrillation

**DOI:** 10.14814/phy2.16131

**Published:** 2024-06-28

**Authors:** Adriano Senter Magajevski, Maria Zildany P. Távora‐Mehta, Niraj Mehta, Débora L. Smith Maluf, Edvaldo C. Pinheiro Silva, Leticia Concato, Marcio Rogerio Ortiz, Eduardo Doubrawa, Marco Stephan Lofrano‐Alves

**Affiliations:** ^1^ Post Graduate Program in Internal Medicine, Internal Medicine Department Federal University of Parana Curitiba Parana Brazil; ^2^ Cardiac Electrophysiology Service of Parana Curitiba Parana Brazil

**Keywords:** atrial fibrillation, head‐up tilt test, hemodynamics, impedance cardiography, peripheral vascular resistance, stroke volume

## Abstract

The hemodynamic response during the transition from the supine to standing position in idiopathic atrial fibrillation (AF) patients is not completely understood. This study aimed to analyze the hemodynamic changes that occur during the head‐up tilt test in idiopathic AF patients. We investigated the hemodynamic changes during the head‐up tilt test with impedance cardiography in 40 AF patients (12 with AF rhythm‐AFr and 28 with sinus rhythm‐AFsr) and 38 non‐AF controls. Patients with AFr had attenuated SVI decrease after standing when compared to AFsr and non‐AF [ΔSVI in mL/m^2^: −1.3 (−3.4 to 1.7) vs. −6.4 (−17.3 to −0.1) vs. −11.8 (−18.7 to −8.0), respectively; *p* < 0.001]. PVRI decreased in AFr but increased in AFsr and non‐AF [ΔPVRI in dyne.seg.m^2^/cm^5^: −477 (−1148 to 82.5) vs. 131 (−525 to 887) vs. 357 (−29 to 681), respectively; *p* < 0.01]. Similarly, compared with non‐AF patients, AFr patients also had a greater HR and greater CI increase after standing. The haemodynamic response to orthostatic challenge suggests differential adaptations between patients with AF rhythm and those reverted to sinus rhythm or healthy controls. Characterizing the hemodynamic phenotype may be relevant for the individualized treatment of AF patients.

## Graphical abstract

We used bioimpedance cardiography to evaluate the hemodynamic response of patients with idiopathic AF compared to normal patients. We demonstrate that a higher percentage of AF patients show a greater drop in PVRI and a smaller drop in SVI than non‐AF patients in the tilt test (upper panel). AF patients reverted to sinus rhythm showed an intermediate response. The lower panel shows the record of the differential behavior of AF and non‐AF patients during the tilt test.

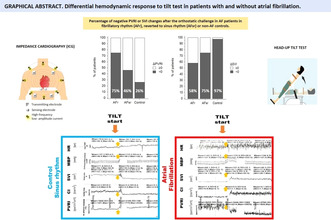

## 1 INTRODUCTION

Atrial fibrillation (AF) is the most common sustained arrhythmia in clinical practice, the prevalence of which is estimated to be 0.5%–1%, and the incidence of AF is constantly increasing due to population aging. It is associated with significant increases in morbidity and mortality, leading to exercise intolerance, tachycardiomyopathy, heart failure, and thromboembolic phenomena (Hindricks et al., 2020). A recent meta‐analysis also demonstrated a correlation between falls and syncope with this arrhythmia (Malik et al., 2020). Several mechanisms contribute to the pathogenesis of this disease, and changes in the autonomic nervous system (ANS) have been implicated as possible triggers for the onset and maintenance of this disease, with adrenergic hyperactivity occurring in 10%–15% of patients with paroxysmal AF (Coumel, 1994; Rocha et al., 2021). Studies using invasive or noninvasive methods have demonstrated that patients with AF exhibit hemodynamic adaptations at rest compared to patients without this arrhythmia, as detected by differences in indices such as heart rate (HR), blood pressure (BP), stroke volume (SV), cardiac output (CO), and peripheral vascular resistance (PVR) (Ding et al., 2016). However, few studies have focused on the transition from the supine to the standing position using the tilt test in AF patients. In most of these studies, the main focus was on indices of autonomic function such as HR and baroceptor reflex variability, and not on hemodynamic indices (Brignole et al., 1993; Cerutti et al., 2014; Hsieh et al., 1998; Ingemansson et al., 1998; Lok & Lau, 1998; Oliveira et al., 2009; Patel et al., 2018; Zuk et al., 2023). The characterization of the hemodynamic adaptations in this scenario could be important for a better understanding of AF pathophysiology, as well as for the diagnosis and management of dysautonomia in these patients. In the present study, using impedance cardiography (ICG) during the head‐up tilt test, we compared the hemodynamic response to orthostatic challenge of AF patients with that of healthy controls.

## 2 MATERIALS AND METHODS

This was a single‐center and cross‐sectional study. We evaluated all consecutive patients who presented to the service with idiopathic AF for cardiology consultation. Patients were invited to undergo a hemodynamic head‐up tilt test at the Cardiac Electrophysiology Service of Parana and the Hospital of Clinics of the Federal University of Parana from January 3, 2012 to December 1, 2021. We included patients with AF without structural heart disease or other known conditions predisposing them to the onset of AF (idiopathic AF). Patients with the following conditions were excluded: hypertension, diabetes mellitus, heart valve disease, previous history of acute myocardial infarction or stroke, left ventricular dysfunction (ejection fraction <54% in women or < 52% in men, obtained by the Simpson method within a maximum period of 6 months before inclusion), cardiomyopathies, thyroid disease, kidney or liver disease, collagen diseases, neurological diseases, rheumatological diseases, sleep apnoea, pregnancy, life expectancy <1 year, use of diuretics or antihypertensive drugs, with the exception of medications used to treat AF (beta‐blockers or calcium channel blockers did not meet the exclusion criteria, but a pause in the medication was performed according to the protocol). Consecutive healthy and asymptomatic volunteers who sought medical support for a check‐up, whose clinical laboratory evaluation was normal, with no history of AF or other comorbidities and whose pathological response did not follow the tilt test, were included in the comparative non‐AF control group. Only one patient in the AF group refused to undergo the tilt test. In the control group, no patient refused to undergo the exam. The Research Ethics Committee of the Federal University of Parana approved the study, which was registered at Plataforma Brasil, the Brazilian database of research records involving human beings, from the Brazilian National Council of Health (registration number CAAE: 13483119.9.0000.0096).

### 2.1 Study protocol

Patients underwent the head‐up tilt test at 70 degrees after 6 h of fasting. All patients are advised to avoid strenuous physical activity and the consumption of tobacco, alcohol, and coffee in the 12 h prior to the test. Patients were assessed regarding the degree of hydration by clinical examination before the tilt test (e.g., mucous membrane hydration, venous turgor). In the case of signs of dehydration, the examination was not performed. After 5 min in the supine position, the patient was placed in the orthostatic position for 20 min. We interrupted the exam at any time in the case of a vasovagal reaction characterized by a decrease in HR and/or BP associated with symptoms of syncope or presyncope. During the examination, the room temperature was set between 23°C and 25°C. We suspended negative chronotropic medications (beta‐blockers, calcium channel antagonists, and antiarrhythmics) for five half‐lives before the tilt test, with the exception of patients for whom the drug was essential for HR control.

To evaluate the hemodynamic variables during the tilt test, we used a commercially available hemodynamic monitor (Task Force Monitor® CNSystems Medizintechnik AG Austria, 2008). A set of four electrocardiogram (ECG) electrodes, 3 band electrodes, and a neutral electrode were fixed to the patient. This monitor integrates a 2‐channel ECG to obtain the R‐R interval and HR variability, continuous BP wave measurements via the vascular unloading technique on the small arteries of the fingers (automatically corrected to oscillometric BP values obtained on the brachial artery at the contralateral arm) and an ICG for hemodynamic variable measurements. To measure the impedance signal, the external electrodes generate an alternating current of high frequency and low amplitude. The internal sensors capture instantaneous changes in voltage that reflect the variation in thoracic impedance due to changes in blood volume within large vessels during systole and diastole. A signal‐processing tool eliminates the influence of breathing on thoracic impedance variation, making it possible to calculate the SV from the thoracic aorta.

Impedance cardiography directly measures certain parameters (such as HR and thoracic fluid content), whereas others are calculated. SV is the main calculated parameter, derived from the measured impedance difference across the thorax. After calculating SV, CO (SVxHR) and total peripheral resistance (TPR = mean arterial BP/CO) are easily determined. Validation studies comparing ICG against traditional invasive and noninvasive methods (pulmonary artery catheterization, Fick method and thermodilution, echocardiography) to determine CO have shown excellent agreement (Bayram & Yancy, 2009; Bloch & Russi, 1997; Fortin et al., 2006; Harford et al., 2019; Osbak et al., 2011; Sageman et al., 2002).

The hemodynamic variables (and their respective indexes, corrected for body surface area) obtained during the tilt test were HR, systolic (SBP), and diastolic (DBP) blood pressure (BP), SV, stroke volume index (SVI), CO, cardiac index (CI), PVR, and peripheral vascular resistance index (PVRI). Total arterial compliance (TAC) was calculated as the ratio of the SV to the pulse pressure (SBP‐DBP) as cited by Medina‐Lezama et al (Medina‐Lezama et al., 2018). We recorded the variables in the supine position and after the orthostatic challenge for a period of 20 min. The device calculates PVR by using the BP formula: MBP = SV × HR × PVR, with PVR = MBP/(SV × HR). CO was calculated according to the formula: CO = HR × SV. The equipment analyzed the mean values of haemodynamic parameters in five traditional periods during the examination: supine position (S); 0–5 (tilt 5′); 5–10 (tilt 10′); 10–15 (tilt 15′), and 15–20 (tilt 20′) minutes of inclination. For a determined variable, we also calculated the change in relation to the supine position (Δ) as the maximum difference during the 20‐min interval in the orthostatic position. We divided the patients into 3 groups according to their heart rhythm during the examination: (1) persistent or permanent AF patients (AF rhythm, or AFr), (2) paroxysmal or reverted AF patients in sinus rhythm during examination (AF sinus rhythm, or AFsr) and non‐AF patients (Control).

### 2.2 Statistical analysis

The data are expressed as medians and interquartile ranges (IQRs) or proportions. We used the D'Agostino–Pearson test to analyze the normality of the distribution of each variable. The random case–control matching procedure was used based on the following criteria: maximal age difference of 2 years; sex, male = 1 or female = 0; use = 1 or not = 0 of medication with a negative chronotropic effect; and BMI categories (<25 = 0; ≥25 and < 30 = 1; ≥30 and < 35 = 2; ≥35 and < 40 = 3; and ≥ 40 = 4). To compare three groups, we used one‐way ANOVA followed by the Tukey–Kramer post hoc test. The comparison of repeated measures was performed by RM‐ANOVA. To analyze the association between two categorical variables, we used the chi‐square test. The associations between a binary dependent variable (AF rhythm = 1 and sinus rhythm = 0) and multiple independent variables were analyzed via logistic regression. We evaluated the relationship between one dependent continuous variable (Y) and one or more independent variables (Xi) by multiple regression. The independent variables for evaluation in the multiple regression model were selected when there was a significant association with the dependent variable in the simple linear regression. Results with *p* values < 0.05 were considered significant. Statistical analysis was performed using MedCalc for Windows, version 18.5 (MedCalc Software, Ostend, Belgium).

## 3 RESULTS

### 3.1 Clinical characteristics of the study population

We included 40 AF patients (28 AFsr and 12 AFr) and 38 non‐AF patients, comprising 78 patients in total (Table 1). The population of AF patients was characterized by older age and male predominance. There was a significantly greater proportion of men in the AFr group than in the non‐AF group (*p* = 0.016), but there was no significant difference between the AFr and AFsr groups or between the AFsr and non‐AF groups. Age was significantly greater in the AFr group than in the non‐AF group. Patients in the AFsr subgroup had a greater BMI than patients in the non‐AF subgroup, but the difference was not significant. The proportion of patients using medications with negative chronotropic or inotropic effects was 41% (5/12) for AFr and 25% (7/28) for AFsr (*p* = NS), while none of the non‐AF were using medications.

**TABLE 1:** Study population.

	AF				
	All AF (*n* = 40)	AFr (*n* = 12)	AFsr (*n* = 28)	Non‐AF (*n* = 38)	*p* Value
Male	30 (75%)	11 (91%)^a^	19 (67%)	20 (52%)	0.040
Female	10 (25%)	1 (8%)	9 (33%)	18 (48%)	
Age (years)	56 (47;63)	58 (56;63)^a^	52 (43;63)	45 (36;54)	<0.001
BMI (Kg/m^2^)	26.4 (25.3;27.6)	24.5 (23.1;26.5)	27.0 (24.4;29.9)^a^	24.2 (21.7;26.2)	<0.001
Medication	12 (30%)	5 (41%)^a^	7 (25%)^a^	0 (0%)	<0.001
Beta‐blocker	9 (22%)	4 (33%)	5 (18%)	0 (0%)	
CCB	2 (5%)	1 (8%)	1 (3.5%)	0 (0%)	
Antiarrhythmics	6 (15%)	2 (16%)	4 (14%)	0 (0%)	

Abbreviations: BMI, body mass index; CCB, calcium channel blocker.

^a^ Significant difference compared to non‐AF.

### 3.2 Clinical and hemodynamic variables in the supine position

**FIGURE 1:**
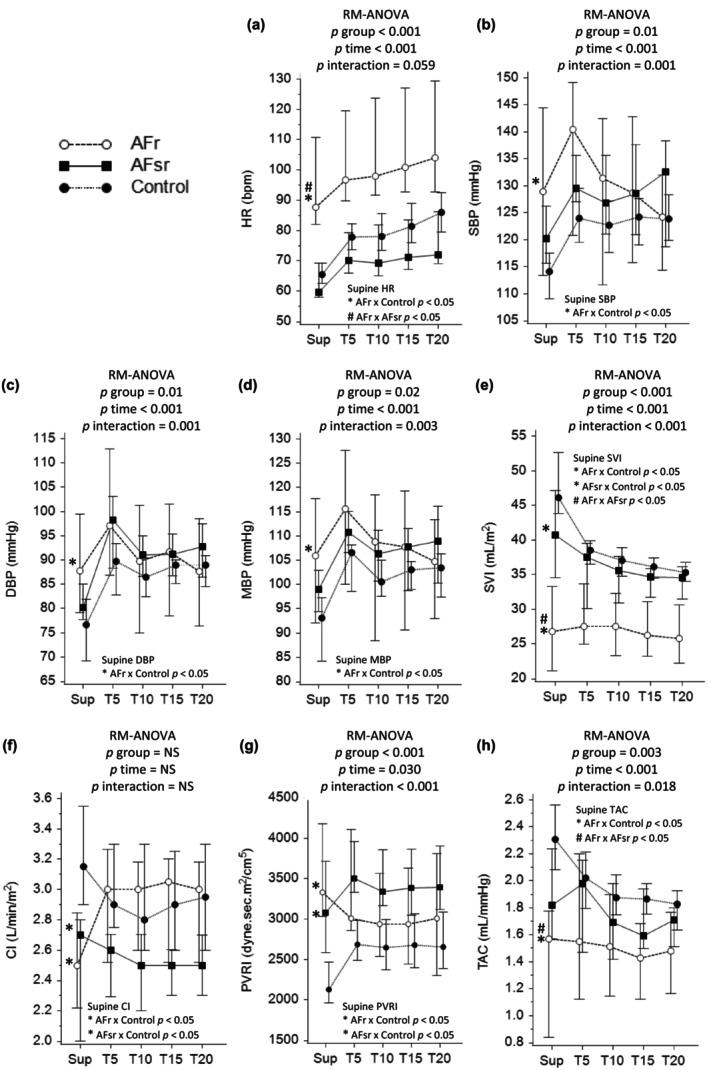
Comparison of hemodynamic parameters between groups in the supine position and at different time points after standing. Data are reported as median and 95% CI. (a) HR, heart rate; (b) SBP, systolic blood pressure; (c) DBP, diastolic blood pressure; (d) MBP, mean blood pressure; (e) SVI, stroke volume index; (f) CI, cardiac index; (g) PVRI, peripheral vascular resistance index; (h) TAC, total arterial compliance; Sup: Supine position; T5, T10, T15 and T20 are measurement time points (in minutes) after standing; *Significant difference compared to the non‐AF; ^#^Significant difference compared to AFsr.

Figure 1 and Table S1 show the data obtained in the supine position and during the head‐up tilt test. In the supine position, the HR was significantly greater in the AFr group than in the AFsr and non‐AF groups. Patients in the AFr group also presented higher SBP and DBP than did non‐AF patients, but these differences were not significant compared to those in the AFsr group. AFr and AFsr had lower SV and SVI values than did non‐AF, and those in AFr had lower SV and SVI values than did those in AFsr. The proportion of patients with abnormally low SVI (<35 mL/m^2^) in AFr was 91.6% (11/12), while AFsr 39.2% (11/28) and non‐AF only 5.2% (2/38) (*p* < 0.001). The CI was lower in AF patients than in non‐AF patients, but there was no difference between AFsr and AFr. The PVR and PVRI were higher in the AF group than in the non‐AF group, but there were no significant differences between the AFsr and AFr groups. The proportion of patients with abnormally high PVRI (>2500 dyn.sec.m^2^/cm^5^) in the AFr group was 91.6% (11/12), while it was 78.5% (22/28) in the AFsr group and only 39.4% (15/38) in the non‐AF (*p* < 0.001). Supine TAC was lower in the AFr group than in the AFsr and non‐AF groups, but there was no significant difference between the AFsr and non‐AF group.

### 3.3 Clinical and hemodynamic variables in the orthostatic position

**FIGURE 2:**
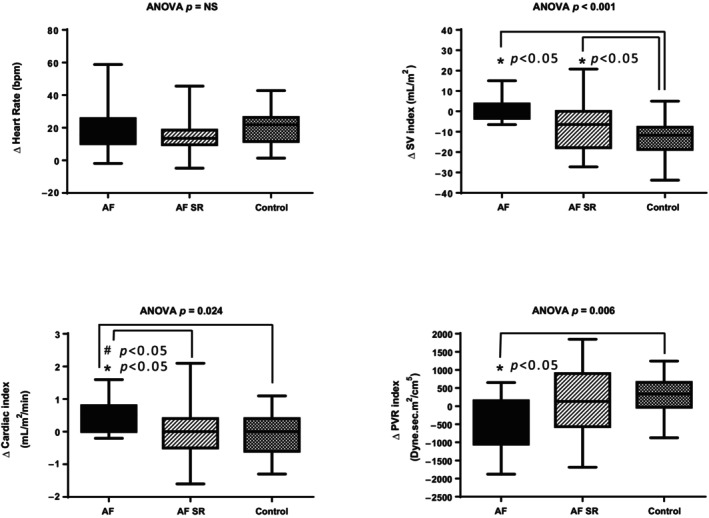
Comparison of the maximal changes (Δ) within 20 min of standing between groups. (a) ΔHR, heart rate; (b) ΔSVI, stroke volume index; (c) ΔCI, cardiac index; (d) ΔPVRI, peripheral vascular resistance index. *Significant difference compared to the non‐AF group; ^#^Significant difference compared to the AFsr group.

After the orthostatic challenge, we observed significant differences in the change rates in SBP, DBP, MBP, SV, SVI, CO, PVR, PVRI, and TAC between the groups (see p interaction in Figure 1 and Table S1). There were no significant differences in the HR change rate or in the ΔHR between the groups. There was a significant difference in ΔMBP between the groups, with AFr patients exhibiting less variation than AFsr and non‐AF patients. In addition to the lower supine SVI, AFr patients had a less pronounced decrease in this variable during orthostatic challenge than did non‐AF patients, but there were no differences in relation to AFsr (Figure 2, right upper panel). The proportions of patients with negative ΔSVIs were 58% (7/12) in the AFr group, 75% (21/28) in the AFsr group, and 97% (37/38) in the non‐AF group (*p* < 0.01). Patients in the AFr group had greater ΔCO and ΔCI values than did non‐AF patients, most likely related to the higher HR and lower decrease in the SVI; moreover, the ΔCO was significantly greater in the AFr group than in the AFsr. The median ΔTAC was negative in the three groups after the orthostatic challenge and was more pronounced in the non‐AF group than in the AFr group.

The AFr group had a negative median ΔPVR and ΔPVRI after standing, which were significantly different from those of the AFsr and non‐AF groups. AFsr and non‐AF patients had positive median ΔPVRs and ΔPVRIs, without significant differences between these groups (Figure 2, right bottom panel). However, the absolute median values for the PVRIs in the AFr group remained greater than those in the other two groups within 20 min of standing, with AFsr maintaining higher absolute median values than non‐AFRs (Figure 1 and Table S1). The proportion of patients with negative ΔPVRIs in the AFr group was 75% (9/12), while it was 46% (13/28) in the AFsr group and only 26% (10/38) in the non‐AF group (*p* < 0.01).

### 3.4 Confounding clinical variables

According to the clinical characteristics presented in Table 1, the AF and control groups differed significantly in terms of age, sex, BMI, and the use or absence of negative chronotropic medications. Figure S1 shows the SVI and PVRI values for the AF and control groups that were matched for these potential confounders. According to these results, the difference observed between AF patients and controls remained when these confounders were controlled for in paired analysis.

### 3.5 SVI and the PVRI relationship

**FIGURE 3:**
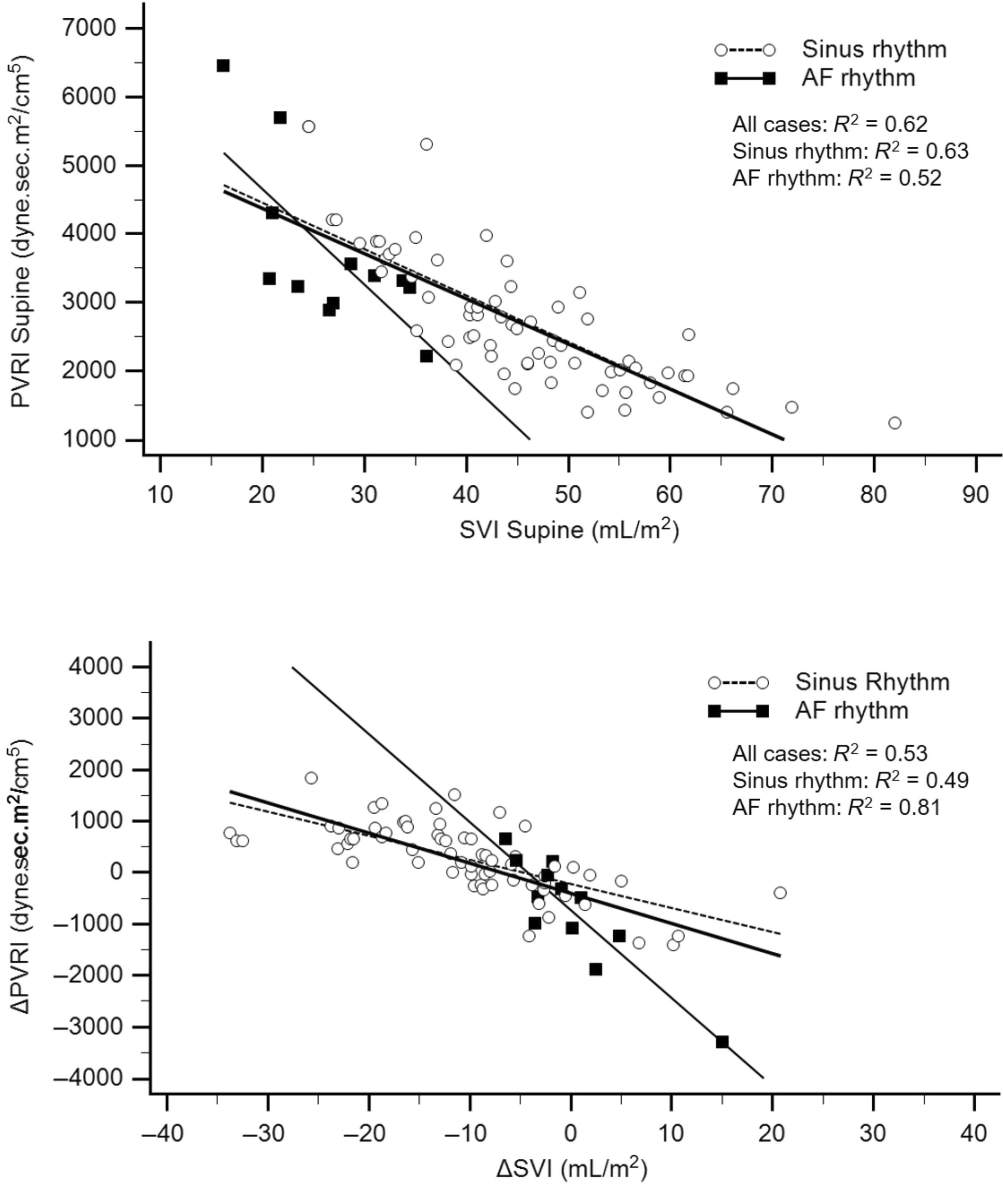
Linear regression and coefficient of determination (*R* ^2^) between supine PVRI and SVI (upper panel) and between ΔPVRI and ΔSVI (bottom panel) for patients in sinus rhythm (empty circles) and AF rhythm (filled squares). The regression equations for the relationship between supine PVRI and SVI in AF rhythm and sinus rhythm patients were PVRI = 7442–139 (SVI) and PVRI = 5811–67 (SVI), respectively, with a significant difference in slope (*p* < 0.05). The regression equations for the ΔPVRI × ΔSVI relationship were ΔPVRI = −727–171 (ΔSVI) for AFr patients and ΔPVRI = −217–46 (ΔSVI) for sinus rhythm patients, with significant differences in terms of comparison of slopes (*p* < 0.001) and intercepts (*p* < 0.001).

Figure 3 shows the inverse relationships, linear regressions and coefficients of determination (*R* ^2^) between the supine PVRI and SVI (upper panel) and between the ΔPVRI and ΔSVI (bottom panel). Compared with those in the other two groups, the slopes in the AFr patients in sinus rhythm were steeper (*p* < 0.001), with greater variation in the ΔPVRI for a given ΔSVI, than those in the AFsr or non‐AF patients (bottom panel).

### 3.6 Determinants of supine SVI and PVRI

Multiple regression analysis discriminated the determinants of supine PVRI and SVI (Table 2). For supine PVRI, the independent determining variables were (in descending order) the supine SVI, MBP, HR, and AF rhythm. The multivariate model including these four variables had a coefficient of determination (*R* ^2^) of 0.87. For the supine SVI, the independent determinants were the supine PVRI, HR, MBP, sex, and supine TAC, with an *R* ^2^ of 0.86. The independent determinants of ΔPVRI according to the multiple regression analysis were, in descending order, supine PVRI, ΔMBP, ΔHR, ΔSVI, age, supine MBP, and BMI. For the ΔSVI, the independent determinants were the ΔTAC, supine PVRI, study group, and supine MBP (Table 3).

**TABLE 2:** Determinants of supine PVRI and SVI by multiple regression analysis.

	Supine PVRI		
Variable	Coef b	*R* semipartial	*p*
Supine SVI	−63.5	0.59	<0.0001
Supine MBP	34.2	0.34	<0.0001
Supine HR	−36.6	0.33	<0.0001
AF rhythm	475.7	0.10	0.013
Model *R* ^2^ = 0.87, *p* < 0.0001			
Variables not included: study group, supine TAC, sex, age, BMI.			

	Supine SVI		
	Coef b	*R* semipartial	*p*
Supine PVRI	−0.01	0.40	<0.0001
Supine HR	−0.41	0.33	<0.0001
Supine MBP	0.26	0.16	<0.001
Sex	−3.56	0.13	0.003
Supine TAC	3.02	0.11	0.011
Model *R* ^2^ = 0.86, *p* < 0.0001			
Variables not included: rhythm, study group, age, BMI.			

Abbreviations: HR, heart rate; MBP mean blood pressure; PVRI, peripheral vascular resistance index; *R* semipartial, the proportion of variance accounted for by the independent variable relative to the total variance of the dependent variable; *R* ^2^, coefficient of determination. Variables not included were those not retained by the multivariate model after stepwise selection; SVI, stroke volume index; TAC, total arterial compliance.

**TABLE 3:** Determinants of ΔPVRI and ΔSVI by multiple regression analysis.

	ΔPVRI		
Variable	Coef b	*R* semipartial	*p*
Supine PVRI	−0.59	0.36	<0.0001
ΔMBP	32.8	0.30	<0.0001
ΔHR	−21.4	0.27	<0.0001
ΔSVI	−28.9	0.23	<0.0001
Age	16.5	0.21	<0.0001
Supine MBP	17.2	0.15	<0.001
BMI	12.3	0.15	<0.001
Model *R* ^2^ = 0.87, *p* < 0.0001			
Variables not included: study group, AF rhythm, sex, supine SVI, supine HR, supine TAC, ΔTAC.			

	ΔSVI		
	Coef b	*R* semipartial	*p*
ΔTAC	9.07	0.44	<0.0001
Supine PVRI	0.004	0.25	<0.001
Study group	2.50	0.15	0.011
Supine MBP	−0.16	0.14	0.018
Model *R* ^2^ = 0.75, *p* < 0.0001			
Variables not included: rhythm, sex, age, BMI, supine HR, supine TAC, ΔFC, ΔPVRI, ΔMBP.			

Abbreviations: BMI, body mass index; HR, heart rate; MBP mean blood pressure; PVRI, peripheral vascular resistance index; *R* semipartial, the proportion of variance accounted for by the independent variable relative to the total variance of the dependent variable; *R* ^2^, coefficient of determination. Variables not included were those not retained by the multivariate model after stepwise selection; SVI, stroke volume index; TAC, total arterial compliance.

### 3.7 Variables associated with AF

We analyzed the association between variables and cardiac rhythm during examination by using stepwise logistic regression (AF rhythm = 1; sinus rhythm = 0). The supine HR and SVI were found to be the independent variables associated with AF rhythm in the multivariate analysis (AUC = 0.98, 95% CI = 0.91–0.99, *p* < 0.0001) (see Table S2). The supine SVI was the only independent variable associated with AF diagnosis (AFr or AFsr = 1 and non‐AF = 0) according to multivariate analysis (AUC = 0.79, 95% CI = 0.69–0.88, *p* < 0.0001) (Table S3).

## 4 DISCUSSION

The main finding in our study using ICG during the tilt test in idiopathic AF patients was the difference in the relationship between the PVRI and SVI in AF patients with a fibrillatory rhythm (AFr) compared to that in AF patients who reverted to sinus rhythm (AFsr) and non‐AF patients.

### 4.1 Hemodynamic findings in the supine position

A distinct characteristic presented by AF patients in our study was the presence of increased PVRIs in the supine position compared to non‐AF patients. Interestingly, not only AF patients in fibrillatory rhythm (AFr) presented high PVRI, but also patients with paroxysmal or reversed AF who underwent the examination in sinus rhythm (AFsr). Furthermore, the proportion of patients with abnormally high PVRIs (>2500 dyn.sec.m^2^/cm^5^) was greater in AFr and AFsr patients than in non‐AF patients (Suehiro et al., 2013).

The older age and greater proportion of male patients in the AF groups may partially explain this difference since increased PVR is related to a greater incidence of AF in elderly men (Dai et al., 2015; Feinberg et al., 1995; Wong et al., 2022).Our data demonstrate that age was higher in AF patients and correlated with AF rhythm during the exam. However, age was not a determinant of the PVRI according to the regression analysis. Sex was associated with the diagnosis of AF but not with the fibrillatory rhythm during the examination. Furthermore, the difference in the PVRI between AF patients and controls persisted when we matched the analysis by age or sex. This finding suggested that the adaptations of PVR are additional to those caused by age and sex and are probably related to other arrhythmia‐linked mechanisms since the presence of a fibrillatory rhythm was a determinant of PVRI.

Another explanation for the greater PVR in AF patients is their greater BMI, since studies also describe the association between these two variables and an increased incidence of AF (Barton et al., 2012; Rosenberg et al., 2012). In our study, AFsr patients showed greater BMI than non‐AF patients. Moreover, logistic regression revealed that BMI was positively correlated with the diagnosis of AF. However, we did not observe a significant association between BMI and AF rhythm during the examination, and BMI was not a determinant of supine PVRI according to the multiple regression analysis. Furthermore, the PVRI (BSA‐indexed PVR) was significantly greater in AF patients than in non‐AF controls, even when the analysis was matched by BMI, suggesting that the high vascular resistance in these patients is likely due to other physiological changes caused by arrhythmia.

Most likely, the higher PVRIs in patients with idiopathic AF are the result of additional neurohormonal adaptations, such as an increased renin‐angiotensin‐aldosterone system (RAAS) and ANS activity; because of elevated HRs, loss of the atrial component of diastolic filling and low SV can occur in these patients (Berkowitsch et al., 2010; Fortin et al., 2006; Gilewski et al., 2013; Hsieh et al., 1998; Lin et al., 2021; Patel et al., 2018). The effects of RAAS activation in promoting vasoconstriction in peripheral arterioles and increasing arterial stiffness and BP are well known (Lage et al., 2022). Increased pulse pressure, reflected by a high “pulsatile load”, leads to atrial distension and is associated with a greater incidence of new AF (Roetker et al., 2014). According to our data, total arterial compliance, as calculated by the SV‐to‐pulse pressure ratio, was reduced in AF patients with a fibrillatory rhythm. Sympathetic activation is also directly related to the increase in the PVRI and BP through the activation of alpha‐1 adrenergic receptors in blood vessels (Bruno et al., 2012).In addition to its hypertensive effect, sympathetic activation is believed to potentiate arrhythmia mechanisms such as automatism and reentry (Arora, 2012; Tisdale et al., 2006).

Our data are in line with those of others demonstrating that AF patients have a reduced SVI (Alboni et al., 1995; Gilewski et al., 2013).In our sample, 91% of the AFr patients and 39% of the AFsr patients had SVIs lower than the normal value (>35 mL/m^2^), compared to only 5.2% of non‐AF controls. These data agree with a recent study in which the SVI was the strongest predictor of recovery of hemodynamic indices and improvement in cardiac function after catheter ablation (Nakatani et al., 2018)We also found that PVRI, HR, MBP, and sex were independent determinants of the supine SVI according to the multiple regression model, in agreement with the findings of other studies (Nakatani et al., 2018).The PVRI and HR have an inversely proportional relationship with the SV. With the absence of effective atrial contractility in AF, part of the left ventricular preload is lost, which reduces the SV. Thus, to maintain MBP, the PVRI and HR must increase. An increased HR is a common finding in AF patients and contributes to shortening diastole and therefore reducing SV. An increased HR also reflects important characteristics of the arrhythmia, such as chaotic atrial activation and low refractory periods of conduction (Daoud et al., 1996).A study involving invasive hemodynamic monitoring elegantly described these adaptations by inducing AF in patients with paroxysmal AF without structural heart disease or hypertension (Alboni et al., 1995).

### 4.2 Hemodynamic findings after the orthostatic challenge

In healthy patients, the main hemodynamic changes within 5 min of standing (compared to those in the supine position) are a reduction of approximately 30% in thoracic blood volume and SV and an increase in HR of 15%–30%, accompanied by a reduction in CO of approximately 20% (Blomqvist & Stone, 2011; de Oliveira et al., 2023; Suojanen et al., 2021; Tavora‐Mehta et al., 2016).In response to these changes, to maintain cerebral perfusion, a series of regulatory cardiovascular mechanisms or reflexes are activated, with the aim of regulating HR, SV (and consequently CO), and PVR, maintaining BP as a controlled variable (Whittle et al., 2022).This standard response was present in our non‐AF group, which showed a 25% reduction in SVI after the orthostatic challenge, which was counterbalanced by a 29% increase in HR, 16% increase in PVRI and 10% increase in MBP, keeping the CI stable.

In contrast, AFr patients exhibited a different response to the orthostatic position. In this group, in fibrillatory rhythm (in which supine PVRI was greater than non‐FA), we observed a reduction in the PVRI instead of an expected increase after the orthostatic challenge. This finding led us to suppose an abnormal or saturated response of alpha1‐adrenergic receptors to sympathetic activation but with maintained beta1‐ and beta2‐adrenergic responses since, in parallel with the reduction in the PVRI, there was an increase in HR similar to that in the other groups. This increase in HR counterbalanced the reduction in the PVRI after standing, which allowed the maintenance of MBP (since MBP=SV × PVR × HR). A lower afterload explained the smaller decrease in the SVI in this group. Other possible explanations for the maintenance of SVI include excessive beta1‐adrenergic stimulation in the presence of an inappropriate alpha‐adrenergic response accompanied by increasing myocardial contractility or beta2‐adrenergic stimulation with consequent increased venous return in response to elevated sympathetic activation (Imai et al., 1978).

However, the variation in the PVRI was heterogeneous in AFr patients, decreasing 75% and increasing 25% of patients, and was associated with an increase in the SVI of 42% and a reduction in 58% of patients, respectively. This finding demonstrated that distinct phenotypes of hemodynamic responses to the orthostatic position depend on the individual characteristics of each patient and may be related to AF rhythm duration and permanence. In a retrospective study, the tilt test response of 31 AF patients was compared to 176 patients in sinus rhythm in order to evaluate differences in HRV between groups. Hemodynamic data obtained by ICG were available for most patients. The results did not show significant differences in the variation of SVI, PVRI, and CI during the tilt test between the groups (Patel et al., 2018). These results differ from those obtained in our study. Nonetheless, this divergence may be related to the differences in methodology and studied populations, since in their study, unlike ours, medications with a chronotropic effect were not suspended to perform the tilt test, 16% of the patients were diabetics, 74% were hypertensive and 6.5% had HF.

Interestingly, in general, the haemodynamic response of AFsr patients to orthostatic challenge was intermediate to that of AFr and non‐AFr patients. Thus, we hypothesized that some physiological adaptations in AF patients are long lasting despite the restoration of sinus rhythm. Alternatively, we speculate that these physiological changes may precede and determine the onset and perpetuation of the arrhythmia.

## 5 CONCLUSIONS

In the present study, we demonstrated that patients diagnosed with idiopathic AF exhibit characteristic hemodynamic adaptations. These adaptations occur in the supine position and after the orthostatic challenge, differentiating AF patients in a fibrillatory rhythm from those reverted to sinus rhythm and non‐AF patients.

## 6 LIMITATIONS

Among the limitations of our study, the observational nature of the study is notable. It is also important to consider the possibility of bias in the selection of patients. We did not analyze the total duration of AF in this sample. We also did not compare the data obtained by ICG with those obtained by other established methods for hemodynamic evaluation. ICG has been questioned for accuracy in determining hemodynamic parameters in certain situations such as acute advanced heart failure, likely due to other causes of increased thoracic fluid (pleural effusion, non‐cardiogenic pulmonary edema, and tricuspid regurgitation) (Kamath et al., 2009). Other possible reasons for inconsistencies in ICG measurements may be related to a stiff aorta due to atherosclerosis (affecting the contribution of the aorta to the bio‐impedance signal) and adequate lead placement (small differences in lead placement can lead to markedly different measurements). However, our population was composed of outpatients, without signs of HF and with preserved LVEF, who were euvolemic during the study. The sample is also composed of patients with low cardiovascular risk, therefore the presence of severe aortic disease being unlikely. Nonetheless, ICG's reproducibility has been shown to be excellent in most studied populations (Treister et al., 2005).

## 7 PERSPECTIVES

Atrial fibrillation is a multifactorial disease with complex pathophysiology and includes several adaptive mechanisms for its genesis and perpetuation. Studies on hemodynamic adaptations in patients with idiopathic AF are rare, and it is necessary to evaluate the effects of AF on cardiovascular physiology, without the specific effects of each associated disease. Understanding these adaptations is important for monitoring and treating dysautonomia in patients with AF. The determination of the PVRI and SVI via the tilt test with ICG could be useful in the evaluation of patients with AF to adequately characterize the hemodynamic phenotype and establish the best individual therapeutic choice. One point to consider would be the use of hemodynamic phenotypes based on SVI and PVRI measurements to select patients for randomized studies testing new interventions for the treatment of AF.

## FUNDING INFORMATION

No funding information provided.

## CONFLICT OF INTEREST STATEMENT

The authors declare that there are no conflicts of interest.

## ETHICS STATEMENT

The study followed the Helsinki declaration for human research. The Research Ethics Committee of the Federal University of Parana approved the study, which was registered at Plataforma Brasil, the Brazilian database of research records involving human beings, from the Brazilian National Council of Health (registration number CAAE: 13483119.9.0000.0096).

## Supporting information


Data S1.
