# Supplementary material for: Differential hemodynamic adaptations to tilt test in patients with idiopathic atrial fibrillation
Source: Physiol Rep. 2024 Jun 28;12(13):e16131. doi: 10.14814/phy2.16131 (PMC11213645; doi:10.14814/phy2.16131)
Supplement: Supplementary file 1 — Data S1. [file PHY2-12-e16131-s001.pdf]

Supplementary Figure 1. Paired analysis of potential confounders.

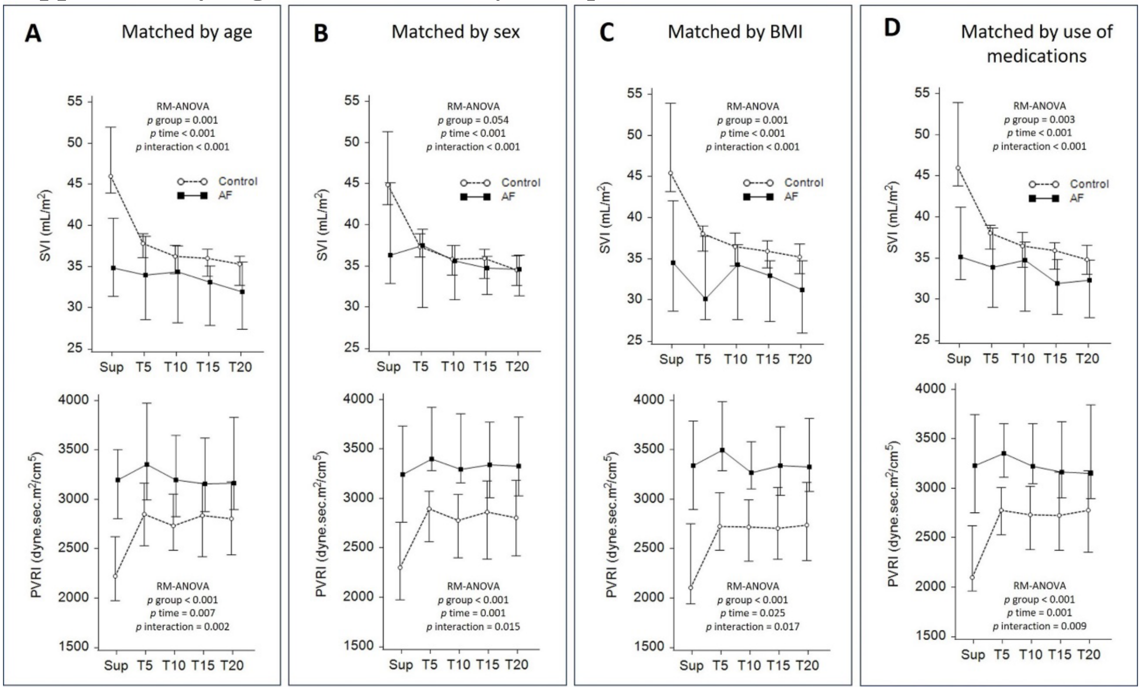

Comparison of SVI (upper panels) and PVRI (bottom panels) between AF (filled squares) and control (empty circles) patients in supine position and in different time points after standing. A. Matched by age in twenty-seven case-control pairs. B. Matched by sex in thirty case-control pairs. C. Matched by BMI in twenty-eight case-control pairs. D. Matched by use or not of negative chronotropic medications in twenty-eight case-control pairs. T5, T10, T15 and T20 are measurement time points (in minutes) after standing position

Supplementary Table 1. Comparative analysis of clinical variables and hemodynamic parameters between AF and non-AF patients.

	**AF**		**Non-AF**	
	AFr (n = 12)	AFsr (n = 28)	(n = 38)	*p* value
HR supine (bpm)	87.6(82.8;109.2)*[#]	60.4 (55.7;71.6)	65.4 (62.0;71.6)	<0.001
HR 5 min	96.6 (90.1;117.7)	71.4 (62.6;83.1)	77.8 (72.8;83.3)	Group: <0.001<br>Time: <0.001<br>Interaction: NS
HR 10 min	98 (92.1;121.7)	70.4 (63.3;85.3)	78.1 (72.5;89.1)	
HR 15 min	100.9 (93.5;127.1)	71.1 (66.6;86.9)	81.5 (73.7;91.9)	
HR 20 min	104.1 (94.1;129.1)	72 (68.2;88.0)	86.0 (77.7;93.2)	
ΔHR 20 min (bpm)	15.0 (12.4;25.6)	12.6 (8.9;17.8)	19.3 (11.3;29.0)	NS
SBP supine (mmHg)	128.9 (114.2;143.5)*	120.6 (112.5;128.7)	114.1 (107.2;119.7)	<0.01
SBP 5 min	140.5 (121.8;148.3)	129.6 (126;138.2)	124.0 (115.5;132.1)	Group: <0.01<br>Time: <0.001<br>Interaction: <0.01
SBP 10 min	131.4 (112.8;141.0)	126.5 (120.7;138.6)	122.7 (115.8;128.7)	
SBP 15 min	128.6 (117.4;138.6)	128.6 (117.6;138.4)	124.2 (118;130)	
SBP 20 min	124.1 (114.4;131.9)	132.6 (118.2;140.2)	123.9 (116.8;129.2)	
ΔSBP 20 min (bpm)	-3.1 (-12.5;7.1)	11.3 (2.0;18.6)	8.9 (3.7;17.4)	NS
DBP supine (mmHg)	87.7 (79.9;98.5)*	80.6 (76.1;89.4)	76.7 (66.2;88.4)	0.001
DBP 5 min	97.0 (87.1;112.8)	96.8 (86.6;104.5)	89.7 (79.5;95.6)	Group: <0.01
DBP 10 min	89.8 (76.2;100.4)	91 (81.9;95.4)	86.5 (78;92.1)	Time: <0.001

DBP 15 min	91.6 (79.6;100.8)	91.2 (82.7;99.9)	89.0 (80;92.6)	Interaction: <0.01
DBP 20 min	87.6 (78.0;96.3)	92.7 (82.6;100.2)	89.0 (79.6;93.1)	
ΔDBP 20 min (bpm)	8.3 (4.7;17.3)	6.5 (0.15;12.8)	12.1 (4.5;17.5)	NS
MBP supine (mmHg)	105.9 (92.3;115.4)*	99 (92.3;104.7)	93 (81.2;99.1)	<0.01
MBP 5 min	115.6 (101.1;127)	110.8 (104.5;117.3)	106.5 (93.8;109.8)	Group: <0.05
MBP 10 min	108.8 (88.8;115.7)	105.9 (95.7;112.6)	100.5 (92.1;106.4)	Time: <0.001
MBP 15 min	107.8 (92.6;116.4)	107.8 (96.2;115.7)	103.1 (94.5;108.2)	Interaction: <0.01
MBP 20 min	104.7 (93.7;113.1)	108.9 (98.1;117.6)	103.4 (95.3;106.6)	
ΔMBP 20 min (bpm)	0.9 (-7.5;6.4)*#	8.9 (3.0;16.6)	9.8 (3.9;15.9)	<0.01
SV supine (mL)	54.0 (42.5;63.0)*#	73.0 (65.0;91.5)*	85.5 (77.0;99.0)	<0.001
SV 5 min	54.5 (49.0;70.0)	68.0 (58.5;74.0)	69.5 (65.0;74.0)	Group: <0.001
SV 10 min	51.5 (48.5;66.5)	68.5 (57.5;73.0)	67.0 (60.0;72.0)	Time: <0.001
SV 15 min	51.0 (46.0;64.5)	66.0 (55.7;74.0)	66.0 (60.0;71.0)	Interaction: <0.001
SV 20 min	49.5 (45.0;63.5)	65.0 (54.2;74.0)	63.0 (58.0;71.0)	
ΔSV 20 min (mL)	-3.0 (-7.0;3.0)*	-13.0 (-30.5;-0.5)	-20.5 (-33.0;14.0)	<0.001
SVI supine (mL/m^2)	26.7 (21.3;32.4)*#	38.5 (32.2;48.6)*	46.1 (42.3;55.6)	<0.0001
SVI 5 min	27.5 (24.9;32.9)	36.7 (29.2;39.8)	38.5 (35.8;41.3)	Group: <0.001
SVI 10 min	27.5 (23.9;31.7)	35.4 (29.6;38.9)	37 (33.8;39.8)	Time: <0.001
SVI 15 min	26.2 (23.5;30.9)	34.7 (28.7;37.7)	36.1 (33.4;39.2)	Interaction: <0.001
SVI 20 min	25.7 (22.6;30.4)	34.6 (28.5;37.1)	35.3 (32.6;38.9)	
ΔSVI 20 min (mL/m^2)	-1.3 (-3.4;1.7)*	-6.4 (-17.3; -0.1)*	-11.8 (-18.7;-8.0)	<0.001
CO supine (L/min)	4.9 (4.4;6.0)	4.7 (4.0;5.5)*	5.8 (4.8;6.6)	<0.01
CO 5 min	6.2 (4.8;6.6)	4.7 (4.3;5.5)	5.4 (4.8;6.6)	Group: <0.01
CO 10 min	6.1 (4.9;6.8)	4.6 (4.3;5.5)	5.2 (4.7;6.1)	Time: <0.05
CO 15 min	6.1 (4.9;6.7)	4.7 (4.4;5.2)	5.2 (4.6;6.0)	Interaction: <0.001
CO 20 min	6.1 (4.9;6.5)	4.8 (4.4;6.7)	5.3 (4.8;6.2)	
ΔCO 20 min (L/min)	0.8 (0.0;1.7)*#	0.0 (-0.9;0.8)*	-0.1 (-1.0;0.7)	<0.05
CI supine (L/min/m^2)	2.5 (2.2;2.7)*	2.6 (2.0;2.9)*	3.1 (2.7;3.8)	<0.001
CI 5 min	3.0 (2.5;3.2)	2.6 (2.2;2.8)	2.9 (2.6;3.4)	Group: NS
CI 10 min	3.0 (2.6;3.1)	2.5 (2.2;2.8)	2.8 (2.5;3.5)	Time: NS
CI 15 min	3.05 (2.5;3.2)	2.5 (2.2;2.7)	2.9 (2.6;3.5)	Interaction: NS
CI 20 min	3.0 (2.5;3.15)	2.5 (2.2;2.8)	2.9 (2.6;3.5)	
ΔCI 20 min (L/min/m^2)	0.45 (0.0;0.8)*	0.0 (-0.5;0.4)	-0.1 (-0.6;0.3)	<0.05
PVR supine (dyne.seg/cm^5)	1616 (1477;2080)*	1671(1266;2016)*	1245 (1012;1470)	<0.001
PVR 5 min	1410 (1366;2115)	1894 (1660;2077)	1464 (1298;1648)	Group: <0.01
PVR 10 min	1345 (1251;1962)	1778 (1610;2005)	1503 (1251;1647)	Time: <0.05
PVR 15 min	1409 (1192;1976)	1762 (1560;2136)	1537 (1301;1658)	Interaction: <0.001
PVR 20 min	1461 (1127;2010)	1810 (1555;2006)	1525 (1257;1684)	
ΔPVR 20 min (Dyn)	-247 (-289;468) *#	71 (-289;468)	180 (-18;411)	<0.01
PVRI supine (dyne.seg.m^2/cm^5)	3332 (3104;3933)*	3109 (2522;3910)*	2126 (1833;2760)	<0.001
PVRI 5 min	3007 (2869;4061)	3447 (3216;4035)	2682 (2300;3074)	Group: <0.001
PVRI 10 min	2934 (2540;3568)	3317 (2944;3897)	2648 (2217;3046)	Time: <0.05
PVRI 15 min	2934 (2461;3622)	3384 (2925;3917)	2681 (2265;3176)	Interaction: <0.001
PVRI 20 min	3007 (2348;4020)	3396 (2893;3796)	2657 (2212;3181)	
ΔPVRI 20 min (dyne.seg.m^2/cm^5)	-477 (-1148;82.5)*#	131 (-525.5;887)	357 (-29;681)	<0.01
TAC supine (mL/mmHg)	1.57 (0.91;1.77)*#	1.81 (1.41;2.46)	2.31 (1.91;2.79)	<0.01
TAC 5 min	1.55 (1.16;2.18)	1.83 (1.45;2.27)	2.00 (1.71;2.29)	Group: <0.01
TAC 10 min	1.51 (1.16;1.86)	1.68 (1.34;1.98)	1.87 (1.68;2.14)	Time <0.001
TAC 15 min	1.42 (1.16;1.68)	1.59 (1.39;2.04)	1.86 (1.68;2.06)	Interaction: <0.05

TAC 20 min	1.48 (1.17;1.74)	1.71 (1.34;2.01)	1.82 (1.60;2.00)	
ΔTAC 20 min	-0.07 (-0.26;0.35)*	-0.2 (-0.91;0.20)	-0.5 (-0.76;-0.18)	<0.05

HR, heart rate; SBP, systolic blood pressure; DBP, diastolic blood pressure; MBP mean blood pressure; SV, stroke volume; SVI, stroke volume index; CO, cardiac output; CI, cardiac index; PVR, peripheral vascular resistance; PVRI, peripheral vascular resistance index; TAC, total arterial compliance. *Significant difference compared to non-AF. # Significant difference compared to AFsr.

Supplementary Table 2. Stepwise logistic regression analysis for association of variables with AF rhythm during examination (AF rhythm = 1 and sinus rhythm = 0).

	Univariate				Multivariate		
Variable	χ^2	OR	95%CI	p	OR	95%CI	p
HR supine	40.3	1.27	1.10-1.48	0.001	1.20	1.04-1.40	0.012
SVI supine	31.9	0.77	0.67-0.89	0.004	0.82	0.67-1.003	0.050
TAC supine	13.8	0.11	0.02-0.47	0.002			
ΔSVI	11.3	1.12	1.03-1.21	0.003			
Age	10.7	1.09	1.02-1.17	0.004			
DBP supine	8.9	1.09	1.02-1.17	0.007			
SBP supine	6.5	1.06	1.01-1.11	0.01			
ΔPVRI	7.9	0.99	0.998-0.999	0.01			
ΔCI	7.8	3.97	1.35-11.5	0.01			
PVRI supine	7.7	1.000	1.0002-1.001	0.007			
ΔTAC	5.9	4.13	1.17-14.5	0.026			
CI supine	4.8	0.34	0.12-0.96	0.04			

HR, heart rate; SVI, stroke volume index; TAC, total arterial compliance; DBP, diastolic blood pressure; SBP, systolic blood pressure; PVRI, peripheral vascular resistance index; CI, cardiac index, OR, odds ratio.

Supplementary Table 3. Stepwise logistic regression analysis for association of variables with the diagnosis of AF (AFr ou AFsr =1 and Non-AF = 0).

	Univariate				Multivariate		
Variable	χ^2	OR	95%CI	p	OR	95%CI	p
PVRI supine	24.3	1.001	1.0007-1.002	<0.001			-
SVI supine	23.8	0.89	0.84-0.94	<0.001	0.89	0.84-0.94	<0.0001
CI supine	18.9	0.20	0.08-0.47	<0.001			-
BMI	13.5	1.08	1.03-1.14	0.001			
ΔSVI	12.9	1.09	1.03-1.15	<0.001			-
Age	11.2	1.06	1.02-1.11	<0.001			-
DBP supine	10.4	1.07	1.02-1.12	0.001			-
SBP supine	8.5	1.05	1.01-1.09	0.003			-
Sex	4.2	0.37	0.14-0.96	0.038			-

PVRI, peripheral vascular resistance index; SVI, stroke volume index; CI, cardiac index; BMI, body mass index; DBP, diastolic blood pressure; SBP, systolic blood pressure; OR, odds ratio.
